# Editorial: Methodological Quality of Interventions in Psychology

**DOI:** 10.3389/fpsyg.2017.00975

**Published:** 2017-06-12

**Authors:** Salvador Chacón-Moscoso, Susana Sanduvete-Chaves

**Affiliations:** ^1^HUM 649—Innovaciones Metodológicas en Evaluación de Programas, Psicología Experimental, Universidad de SevillaSevilla, Spain; ^2^Departamento de Psicología, Universidad Autónoma de ChileSantiago, Chile

**Keywords:** methodological quality, interventions, psychology, design, measurement, analysis

The need to evaluate intervention programs rigorously in different areas of psychology (e.g., health, education, sports, or social welfare) is widespread. However, we find clear methodological weaknesses in professional practice when it comes to evaluating intervention programs.

In many cases, fundamental details are not learned, such as how an intervention is framed, how it was implemented, what aspects of it are responsible for the effects, and how effective it is relative to other alternatives. Such absences hinder the replicability of interventions, learning what program aspects could be improved and how the knowledge from a single intervention can be integrated with other findings. All this prevents the growth of cumulative knowledge, the ability to use research to inform policy, and even the advancement of science.

According to previous research, much of this methodological weakness can be attributed to two factors: disagreement about how to conceptualize and measure methodological quality in evaluation, and the context dependency of existing instruments that claim to measure such quality.

The concept *quality* is complex and multidimensional. It has been defined from different theoretical perspectives that variously emphasize individual concepts or sets of concepts dealing with, for example, internal, external, and construct validity. This theoretical diversity leads to different approaches to measuring research quality, such as scales (tools where at least content, construct, and criterion validity evidence was tested), checklists (tools that have not been tested through an extensive validation process), and general recommendations (taking the form of advice).

The second methodological weakness stems from the context dependency of the instruments used that reduces the chance of the information they generate to be general. Indeed, many tools are used on just one occasion, and so dependable knowledge about its psychometric properties, including reliability and validity, are rarely available.

In this Research Topic, some works present methodological approaches to enhance the quality of psychological intervention, being context independent solutions. Thus, Chacón-Moscoso et al. (a) systematize and summarize the available literature about methodological quality in primary studies to describe the state of the art in assessing the methodological quality of interventions; (b) propose a specific, parsimonious, context independent, 12-items checklist to empirically define the methodological quality of primary studies based on a content validity study; and (c) present an inter-coder reliability study for the resulting 12 items.

Holgado-Tello et al. use Structural Equation Modeling (SEM) as a first approximation to operationalize the analytical implications of threats to validity in quasi-experimental designs. The study presents this empirical solution to the existing weak link between design features, measurement issues, and concrete impact estimation analyses. Finally, Manolov et al. make practitioners and applied researchers aware of the available appropriate options for extracting maximum information from the data. Concretely, they suggest that the evaluation of behavioral change should include visual and quantitative analyses, complementing the substantive criteria regarding the practical importance of the behavioral change.

In a complementary way, this Research Topic also presents original work in different areas where methodological quality has been better assessed in order to estimate unbiased effect sizes and study possible moderator variables influencing the results obtained.

In health area, Cano-García et al. evaluate formatively (before, during and after the intervention), a program of multicomponent psychological intervention for patients with chronic pain implemented: (a) based on techniques with empirical evidence, but developed in Spain; (b) at a public primary care center; (c) among patients with limited financial resources and lower education; (d) by a novice psychologist; and (e) taking measures of all domains of painful experience using the instruments recommended by the Initiative on Methods, Measurement, and Pain Assessment in Clinical Trials (IMMPACT).

Additionally, Moreno et al. use the adversity level associated with family functioning and the positive adaptation level, as measures of a global health score, to distinguish four groups within adolescents: maladaptive, resilient, competent, and vulnerable. Such groups are compared in a number of demographic, school context, peer context, lifestyles, psychological, and socioeconomic variables, which can facilitate or inhibit positive adaptation in each context. In this way, they offer very valuable information for optimizing design and assessment of interventions and policies aimed at fostering adolescent health.

Furthermore, Vargas et al. use animal models of mental illness as a useful tool to characterize indicators of possible cognitive dysfunctions in humans. In this way, the subjectivity of the classical psychological evaluation processes where the patient must calibrate the magnitude of his/her symptoms and therefore the severity of his/her disorder, is overcome.

In education, Liu et al. extend the measurement part of latent transition analysis to the growth mixture model to examine the reading ability development of children. They found that the new model fitted the data well. Results also revealed that most of the children stayed in the same ability group with few cross-level changes in their classes. Finally, after adding the environmental factors as predictors, analyses showed that children receiving higher teachers’ ratings, with higher socioeconomic status, and of above average poverty status, would have higher probability to transit into the higher ability group.

In sports area, Liu et al. examine relevant randomized controlled trials (RCTs) published in the past 20 years (1996–2015) for methodological concerns arise from Lord's paradox. Their analysis revealed that RCTs supporting the positive effect of exercise on cognition are likely to include Type I Error(s). This result can be attributed to the use of gain score analysis on pretest-posttest data as well as the presence of control group superiority over the exercise group on baseline cognitive measures. To improve accuracy of causal inferences in this area, analysis of covariance on pretest-posttest data is recommended under the assumption of group equivalence.

Finally, referring to social welfare, Izquierdo-Sotorrío et al. explore the informant effect and incremental validity to examine the relationships between perceived parental acceptance and children's behavioral problems (externalizing and internalizing) from a multi-informant perspective.

## Author contributions

The two authors contributed to documenting, designing, drafting, and writing the manuscript, and revised it for important theoretical and intellectual content. Additionally, both authors provided final approval of the version to be published and agree to be accountable for all aspects of the work in ensuring that questions related to the accuracy or integrity of any part of the work are appropriately investigated and resolved.

### Conflict of interest statement

The authors declare that the research was conducted in the absence of any commercial or financial relationships that could be construed as a potential conflict of interest.

